# Meta-Analysis Based on Clinical RCTs: The Effect of Molecular Epimerism on the Safety of Glycyrrhizic Acid

**DOI:** 10.1155/2020/3869698

**Published:** 2020-11-30

**Authors:** Hongdou Chen, Fangfang Zheng, Menglei Wang, Xu Wang, Qingqing Yang, Lu Ye, Yao Lu, Shule Yu, Wei Li

**Affiliations:** ^1^Department of Pharmacy, Suqian People's Hospital of Nanjing Drum Tower Hospital Group, Suqian 223800, Jiangsu, China; ^2^Department of Pharmacology, Suqian Hospital Affiliated with Xuzhou Medical University, Suqian 223800, Jiangsu, China

## Abstract

**Objective:**

To carry out the meta-analysis on the clinical safety of glycyrrhizic acid and the influencing factors between 18*α*-glycyrrhizinate (18*α*-GL) and 18*β*-glycyrrhizinate (18*β*-GL).

**Methods:**

Magnesium isoglycyrrhizinate injection was used as the representative preparation of 18*α*-GL, and compound glycyrrhizin injection was used as the representative preparation of 18*β*-GL. The clinical control trial of magnesium isoglycyrrhizinate injection and compound glycyrrhizin injection was searched in a computer, which was published from January 2006 to December 2019 on the databases such as PubMed, China National Knowledge Infrastructure (CNKI), China Science and Technology Journal Database (CSTJ), and Wanfang Medical Network (Wanfang Data). The data associated with adverse drug reactions (ADRs) were extracted. RevMan5.3 was used for statistical analysis.

**Results:**

Finally, 24 studies were included, and 2757 patients were involved, of which the experimental group was mainly treated with magnesium isoglycyrrhizinate, while the control group was mainly treated with compound glycyrrhizin. The results showed that the occurrence of ADRs was significantly lower in the experimental group than that in the control group, and the difference between two groups was statistically significant (RR = 0.26, 95% CI = (0.18, 0.38), *P* < 0.00001). There was no heterogeneity among the studies (*I*^2^ = 0%, *P*=1.00).

**Conclusion:**

Compared with 18*β*-GL, 18*α*-GL had a lower incidence of adverse reactions and better clinical safety.

## 1. Introduction

Glycyrrhizin (GL) is one of the active ingredients in licorice, and it has been widely used in clinical diseases of abnormal liver function. Based on epimerism, GL is classified into 18*α*-glycyrrhizinate (18*α*-GL) and 18*β*-glycyrrhizinate (18*β*-GL) [1]. The difference between the efficacy and safety of these two epimers has become the focus of clinical attention. Currently, the representative 18*α*-GL drug on the market is magnesium isoglycyrrhizinate (MI), and the representative 18*β*-GL drug is compound glycyrrhizin (CG). Existing studies with small samples have shown that MI and CG have differing conclusions of the adverse drug reactions (ADRs). The studies of Ye et al. [2] and Mao et al. [3] showed that there were no differences in ADRs between these two drugs. Many other controlled studies only reported the number of ADR cases from these two drugs and did not perform statistical analyses. Animal studies have shown that the two isomers are different in cortisol competition for 11*β*-hydroxysteroid dehydrogenase [4]; conversely, 11*β*-hydroxysteroid dehydrogenase was directly associated with pseudo-aldosteronism-related symptoms. However, whether the difference in the structure of these two preparations has led to the occurrence of ADR difference has not been reported clinically. Therefore, in this study, meta-analysis was used to explore their relationship of ADRs and 11*β*-hydroxysteroid dehydrogenase in MI and CG in order to provide reference for clinical safe medication and new production preparations.

## 2. Materials and Methods

### 2.1. Literature Retrieval

With the keywords of MI and CG, the literature from January 2006 to December 2019 was searched in the databases such as PubMed, China National Knowledge Infrastructure (CNKI), China Science and Technology Journal Database (CSTJ), and Wanfang Medical Network (Wanfang Data). And then, the references of the literature that met the inclusion criteria were carefully read to obtain the wanted articles. The selected literature was published in Chinese or English. This paper was retrieved by two researchers (Fangfang Zheng and Xu Wang).

### 2.2. Selection Criteria

#### 2.2.1. Literature Inclusion Criteria

1. The subjects were patients with abnormal liver biochemical indicators
2. The case-control study on the experimental group treatment with MI injection and control group treatment with CG injection and other treatment measures in these two groups which were the same in patients with abnormal liver function was analyzed
3. These articles reported in detail the number and presentation of ADRs in the experimental and control groups

#### 2.2.2. Literature Exclusion Criteria

1. The same article is in different databases
2. Different articles using the same data repeatedly
3. ADR data are in doubt

### 2.3. Literature Screening

Two researchers (Lu Ye and Qingqing Yang) extracted the papers. First of all, through reading the title and abstract of the literature retrieved, articles of magnesium isoglycyrrhizinate and compound glycyrrhizin were screened out. Articles that remained after the preliminary screening were screened again by reading the entire articles based on the provided ADR data. Finally, data extracted from all articles were compared to remove duplicate articles. Articles that only reported the number or the presentation of ADRs or articles in which the reported number did not match with the presentation were excluded.

### 2.4. Data Extraction and Quality Evaluation

All the following information was extracted from the included literature: diagnosis, diagnosis and treatment cycle, MI dose (mg), MI origin, CG dose (mg), CG origin, MI sample size, CG sample size, MI-ADR number and presentation, and CG-ADR number and presentation. The quality of the literature was evaluated according to the Cochrane risk-of-bias tool [5]. Low-quality or missed information literature studies were removed. Two evaluators evaluated the literature independently and cross-checked. When there were divergences, the consensus was reached through discussion.

### 2.5. Statistical Analysis

A meta-analysis was carried out by RevMan5.3 software. The publication bias was tested by Stata software. The development of ADRs was evaluated using the relative risk (RR) and 95% confidence interval (CI). Heterogeneity was examined using a homogeneity test (chi-square test). *I*^2^ ≤50% indicated that the heterogeneity might not be important or that there was moderate heterogeneity. The fixed-effect model was used for the meta-analysis. *I*^2^ >50% indicated that heterogeneity was evident. If interference could not be excluded, the random-effect model was used for analysis.

## 3. Results

### 3.1. Literature Retrieval Results

**Figure 1:**
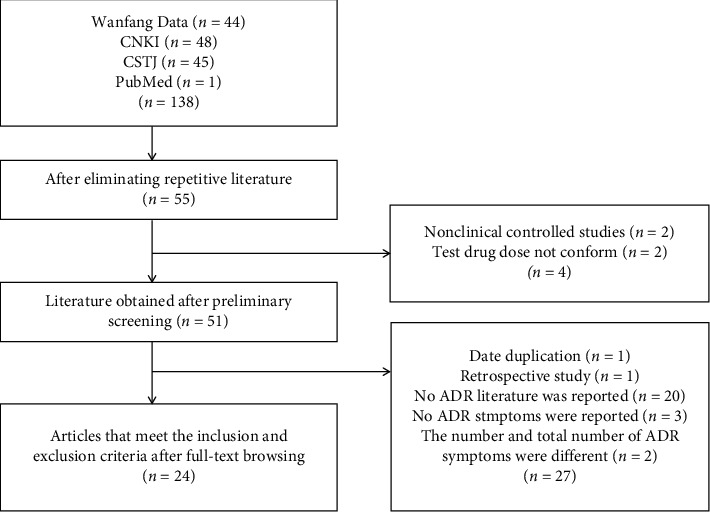
Flow diagram of assessment of studies.

Databases were searched comprehensively, and then, the retrieval results were cross-checked (Figure 1). At the end, 55 articles were chosen for the meta-analysis. Preliminary screening was performed on the titles and abstracts, and two non-RCTs [6, 7] and two articles with nonmatched drugs were removed [8, 9]. After reading the entire articles, one article with repeated data [10], one retrospective study [11], 20 articles that did not report ADR data [12–31], three articles that only reported the number of ADRs but did not report symptoms [32–34], and two articles that had inconsistent ADR numbers and symptoms were excluded [35, 36]. Finally, 24 articles that conformed to the inclusion criteria were included in this study [3, 37–59].

These 24 articles involved a total of 2,757 patients with 1,520 cases in the MI group and 1,237 cases in the CG group. The MI doses in these studies ranged from 50 to 200 mg, and the CG doses ranged from 60 to 200 mg. The MI preparations in these studies were from original manufacturers. Nine studies had labeled the origins of the CG injections, and five studies did not indicate the origin; therefore, a statistical analysis could not be performed (Table 1).

### 3.2. Evaluation of Literature Quality and the Heterogeneity Test

**Figure 2:**
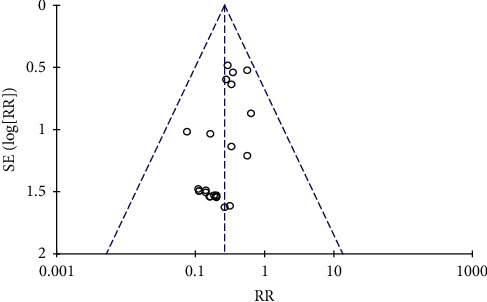
Evaluation of the publication bias of the articles.

**Figure 3:**
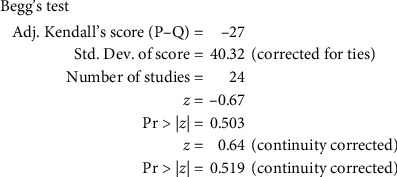
Begg's test.

**Figure 4:**
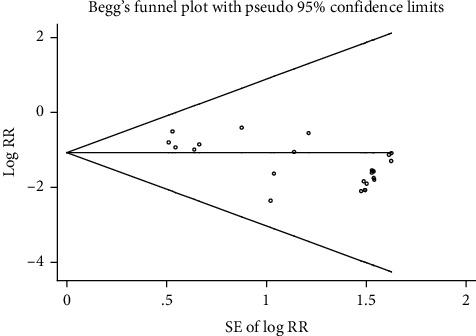
Begg's funnel plot with pseudo 95% confidence limits.

**Figure 5:**
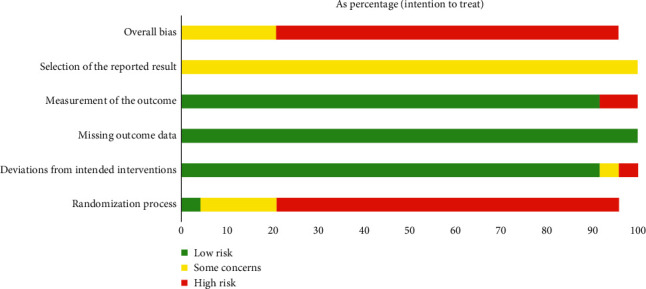
Results of the quality evaluation of the included articles.

Three [37, 38, 57] of the 24 articles described the method of randomization. None of the articles mentioned allocation concealment. One article used a double-blinded method. One article was published in a Chinese-English journal [3], and the other articles were all published in Chinese journals. The determination of ADRs was based on clinical symptoms and laboratory biochemical indicators; therefore, it was objective. Data in all articles were complete, and there was no loss to follow-up. Because most of the articles were published in Chinese journals, publication bias was evaluated using a funnel plot. The results are shown in Figure 2. The funnel plot was tested by Begg's test (Figure 3). The results showed that *z* = 0.64 (continuity corrected), pr > |*Z* | = 0.519 (continuity corrected), and 0.519>0.05, indicating that the results were not statistically significant. All of them are uniformly distributed in Figure 4. Therefore, no publication bias was found by Begg's test. The results of the literature quality evaluation are shown in Figure 5.

### 3.3. The Conditions of ADR Development

The specific conditions of the number and symptoms of ADRs in the articles are shown in Table 2.

**Figure 6:**
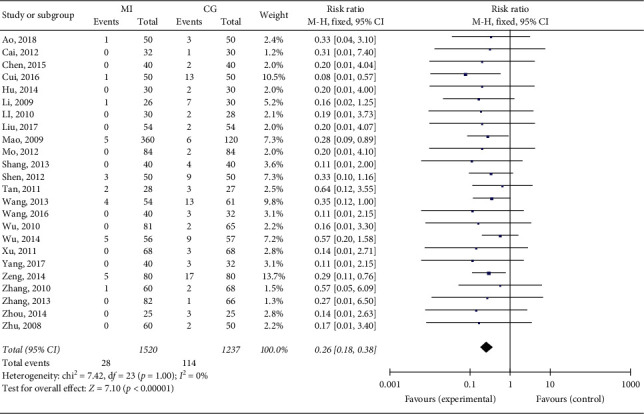
Results of the meta-analysis of ADR development.

The overall analytic results of the 24 studies are shown in Figure 6. They all reported ADR development in both the MI group and the CG group (*n* = 1520/1237). To assess ADR development, the meta-analysis was performed using the fixed-effect model because the heterogeneity among all studies might not be important (*I*^2^ ≤ 50%). The results showed that the RR of ADR development in the MI group was significantly lower than that in the CG group (RR = 0.26, 95% CI = (0.18, 0.38), *P* < 0.00001).

## 4. Discussion

The mechanism of ADR development is very complicated. Molecular heterogenicity is one of the reasons for the difference ADR occurrence in glycyrrhizin preparations. MI is a single-ingredient preparation, in which the non-8*α*-GL content is only 1%. CG, as a representative drug of second-generation glycyrrhizic acid, mainly contains 18*β*-GL and a small amount of 18*α*-GL, and the proportion of 18*β*-GL ranged between 95% and 99%. This study compared the high-purity 18*α*-GL preparation of MI with the clinically extensively used CG preparation that mainly contained 18*β*-GL. Through a comprehensive analysis of the conditions of ADR development in many studies, the results showed that these two preparations had significant differences in the ADR.

### 4.1. Clinical Presentations of ADRs

The statistical analysis of this study showed that the ADRs of GL primarily manifested as lower limb edema, facial edema, increased blood pressure, palpitation, and dizziness and headache. A total of 112 cases accounted for 78.87% of the 142 ADR cases. These frequent ADRs were all associated with pharmacological and toxicological effects of GL and were pseudo-aldosteronism reactions. Pseudo-aldosteronism reactions accounted for 67.86% of ADRs in the MI group and 81.58% of ADRs in the CG group. With MI as the 18*α*-GL preparation, pseudo-aldosteronism was still the main type of ADR reported; however, its incidence was lower than that from the CG presentation, which mainly contained 18*β*-GL. These results were consistent with the study of Li et al. [60].

### 4.2. The Mechanism of Differences

The current view considers that the pseudo-aldosteronism reaction of GL is associated with the drug itself or its metabolic product [61]. Different structures of GL also have metabolic products with different structures in the body. The metabolic product of 18*α*-GL is 18*α*-glycyrrhetinic acid, and the metabolic product of 18*β*-GL is 18*β*-glycyrrhetinic acid. Existing preclinical studies have shown that the incidence of pseudo-aldosteronism reactions produced by 18*α*-glycyrrhetinic acid was lower than that produced by 18*β*-glycyrrhetinic acid. A possible reason is that 18*α*-glycyrrhetinic acid selectively inhibits type 1 11*β*-hydroxysteroid dehydrogenase, whereas 18*β*-glycyrrhetinic acid has inhibitory effects on both type 1 and type 2 11*β*-hydroxysteroid dehydrogenase [4]. These results suggest that differences in the clinical ADRs between these two preparations might also be caused by differences in their molecular conformation. The specific clinical, pharmacological, and toxicological mechanisms and the strength of action require further experimental studies.

### 4.3. The Influence of the Experimental Design Program

The meta-analysis results were influenced by the quality and number of patients in the included articles. None of the articles included in this study described allocation concealment, which might influence the subjective judgments of doctors and patients, particularly the subjective symptoms of ADRs such as dizziness, headache, and palpitation.

## Figures and Tables

**Table 1:** Basic information of included studies.

Study	MI			CG			SI
	Number	Dose (mg)	ADR	Number	Dose (ml)	ADR	
Ao, 2018	50	100	1	50	80	3	③④⑤
Cai, 2012	32	150	0	30	60	1	①②③④⑤
Chen, 2015	40	150	0	40	80	2	①②③④⑤
Cui, 2016	50	150	1	50	80	13	④⑤
Hu, 2014	30	100	0	30	100	2	①②③④⑤
Li, 2009	26	100	1	30	60	7	④⑤
Li, 2010	30	100	0	28	40	2	①②③④⑤
Liu, 2017	54	150	0	54	80	2	①②③④⑤
Mao, 2009	360	100–150	5	120	60	6	①②④⑤
Mo, 2012	84	100–200	0	84	40–60	2	①②③④⑤
Shang, 2013	40	200	0	40	100	4	①②③④
Shen, 2012	50	200	3	50	80	9	①③④⑤
Tan, 2011	28	100	2	27	80	3	①②③④
Wang, 2013	54	150	4	61	80	13	①②④
Wang, 2016	40	150	0	32	80	3	①②③④⑤
Wu, 2010	81	150–100	0	65	80–40	2	①②③④
Wu, 2014	56	150	5	57	80	9	①②④
Xu, 2011	68	150	0	68	100	3	①②③④⑤
Yang, 2017	40	100	0	32	80	3	①②③④
Zeng, 2014	80	50	5	80	30	17	④⑤
Zhang, 2010	60	100	1	68	80	2	④⑤
Zhang, 2013	82	100	0	66	60	1	①②③④
Zhou, 2014	25	200	0	25	100	3	①②③④⑤
Zhu, 2008	60	150	0	50	60	2	①②④

SI: study index; ①: ALT; ②: AST; ③: T-Bil; ④: adverse effects; ⑤: curative effect.

**Table 2:** The presentation and number of ADRs in the MI and CG groups.

ADR presentation	MI group		CG group	
	Number of ADRs	Proportion (%)	Number of ADRs	Proportion (%)
Lower limb edema	3	10.71	23	20.18
Dizziness, headache	6	21.43	21	18.42
Increased blood pressure	2	7.14	20	17.54
Palpitation	5	17.86	13	11.40
Facial edema	3	10.71	16	14.04
Gastrointestinal reaction	5	17.86	8	7.02
Decreased muscle strength	2	7.14	7	6.14
Hypokalemia	1	3.57	3	2.63
Hyperkalemia	0	0.00	0	0.00
Urine occult blood	0	0.00	1	0.88
Facial flushing	0	0.00	1	0.88
Skin rash	1	3.57	1	0.88
Total	28	100.00	114	100.00

## Data Availability

The datasets used and/or analyzed during the present study are available from the corresponding author upon reasonable request.

## Supplementary Materials

Supplementary Materials PRISMA statement and evaluation of publication bias of the articles are provided.
